# Supplementary material for: Modeling strict age-targeted mitigation strategies for COVID-19
Source: PLoS One. 2020 Jul 24;15(7):e0236237. doi: 10.1371/journal.pone.0236237 (PMC7380601; doi:10.1371/journal.pone.0236237)
Supplement: S1 File — (PDF) [file pone.0236237.s001.pdf]

S1 Contact matrix processing

The matrices provided in [23] are asymmetric. There are two important reasons for the asymmetry; the first is simply that each contact reports contacts experienced with other people, and, e.g., the pointee may not be part of the survey population. The second is that the matrices correspond to absolute frequencies of interactions between age groups, rather than frequencies relative to the sizes of the age groups. (In particular, if all groups were equally likely to interact, [23] would report a contact matrix in which each row was equal to the population-proportion vector for the age groups). The dynamics we describe in (1), etc., already account for the proportion of the susceptible population which belongs to each age group, thus we process the contact matrix to correct for the second source of asymmetry by dividing column by the proportion of the population in the corresponding age group. This results in a nearly symmetric matrix, which we symmetrize by taking the average of pairs of elements reflected across the diagonal, averaging out the first source of asymmetry.

The contact matrices reported in [23] have 75-79 as their last age bracket. In our model we give the 80+ age group the same contact patterns as the 75-79 group but right-shifted by 5 years; e.g., after adjusting for population size, people in the 80+ age group interact with people in the 75-79 age bracket at the same rate that people in the 75-79 bracket interact with people in the 70-74 bracket. We use the right shift instead of a direct copy because the right-shift of the 70-74 bracket correlates better with 75-79 bracket than does the 70-74 bracket itself—Pearson correlation .85 versus .32—and this is true even ignoring diagonal entries.

The explicit values of the resulting symmetric contact matrix (before adjustment for a particular R_0 value) are shown in Table S1.

Note that one could use location-specific contact matrices (for work interactions, home interactions, *etc*) as provided in [23], but we have eschewed this choice for our modeling, since this would depend on an accurate model of how these different types of interactions have contributed to reported R_0 values.

Our population figures by age for the United States are those provided for 2019 by the U.S. Census Bureau at
`https://www.census.gov/topics/population/age-and-sex/data/tables.html`.

S2 Contact matrices from mitigations

As discussed in the main text, we model mitigations by reducing transmission rates to, from, and within a population subject to mitigations by 50%, 70%, or 90%, respectively. The population subject to mitigations is either everyone under an age threshold (40, 50, or 60), or 2/3 of those people. In the latter case, a contact matrix is generated by splitting the age groups below the threshold into two populations (the part subject to mitigations and the part which is not). The resulting contact matrices for the various mitigation scenarios are shown in in Fig S1.

S3 Sensitivity of analysis

In Section 4.2 we presented scenarios of age-targeted mitigations where $R_0 = 2.7$ and transmission levels for the groups subject to mitigations are reduced by 70%. In Figures S6, S7, S8, S9, S10, S11, S12, S13, S14 we show scenarios where R_0 is 2.4, 2.7, or 3.0, and the transmission reduction for the group subject to mitigations is 90%, 70%, or

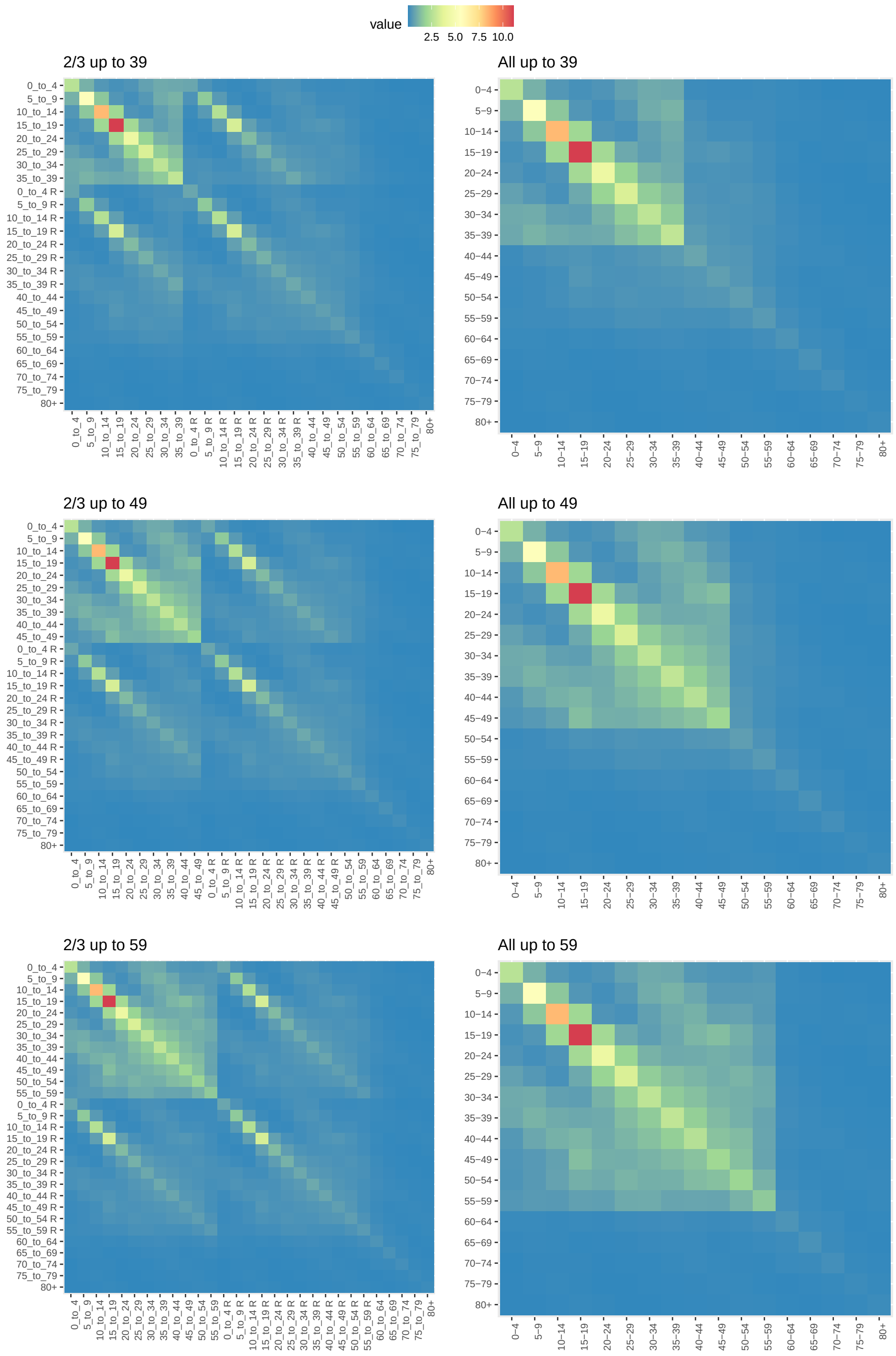

Fig S1. Contact matrices corresponding to the 70% mitigation scenarios from the main text. The left column scenarios correspond to strategies where only 2/3 of the younger age group is released from mitigations. For these scenarios, brackets with the R suffix correspond to the 1/3 fraction of the age-group still subject to restrictions. Note that mitigations are modeled by scaling any entry of the matrix from Fig 1 which lies in a row or column of a group subject to mitigations to 30% of its original value.

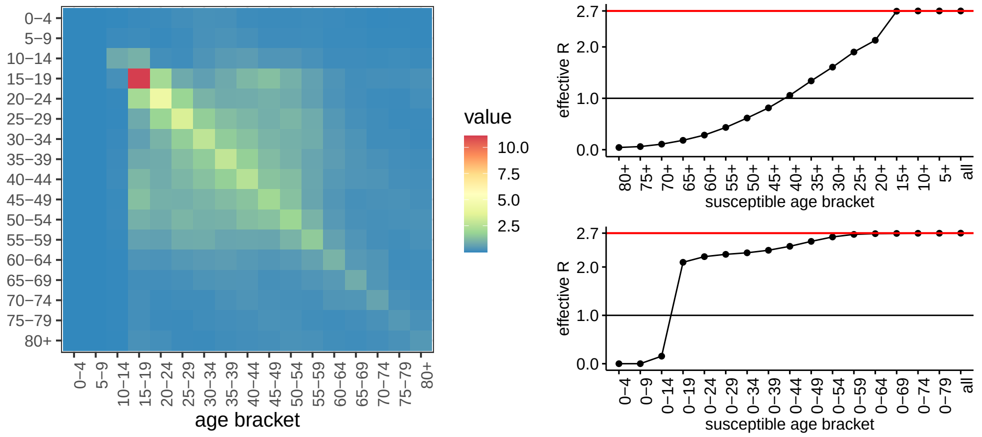

Fig S2. Left: A modified contact matrix for the scenario where children under 15 are 50% less susceptible and 90% less infectious than older age groups. **Right:** Even after rescaling to correspond to $R_0 = 2.7$, we see that older age groups are still much less capable of sustaining an epidemic on their own than are younger age groups.

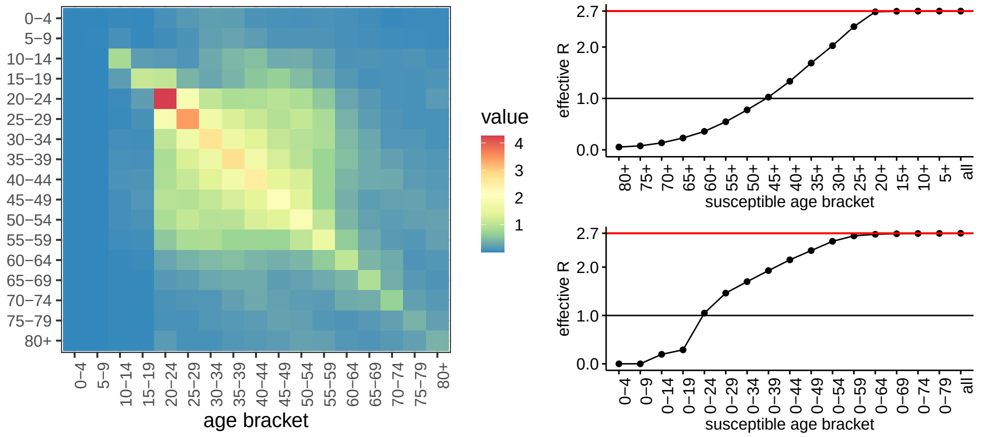

Fig S3. Left: A modified contact matrix for the scenario where children under 20 are 50% less susceptible and 90% less infectious than older age groups. **Right:** Even after rescaling to correspond to $R_0 = 2.7$, we see that older age groups are still less capable of sustaining an epidemic on their own than are younger age groups.

	0-4	5-9	10-14	15-19	20-24	25-29	30-34	35-39	40-44	45-49	50-54	55-59	60-64	65-69	70-74	75-79	80+
0-4	2.60	1.05	0.40	0.24	0.34	0.65	0.87	0.83	0.42	0.35	0.32	0.40	0.31	0.21	0.09	0.12	0.12
5-9	1.05	5.39	1.56	0.39	0.20	0.43	0.91	1.08	0.79	0.45	0.46	0.47	0.30	0.28	0.19	0.16	0.12
10-14	0.40	1.56	8.28	2.02	0.34	0.26	0.62	0.89	1.03	0.66	0.68	0.46	0.22	0.23	0.19	0.24	0.16
15-19	0.24	0.39	2.02	11.11	2.12	0.83	0.57	0.83	1.15	1.33	0.98	0.62	0.30	0.15	0.19	0.18	0.24
20-24	0.34	0.20	0.34	2.12	4.26	1.88	1.07	0.88	0.89	0.99	0.88	0.59	0.27	0.17	0.10	0.09	0.18
25-29	0.65	0.43	0.26	0.83	1.88	3.41	1.69	1.31	1.13	0.95	1.10	0.86	0.39	0.20	0.13	0.09	0.09
30-34	0.87	0.91	0.62	0.57	1.07	1.69	2.72	1.63	1.38	1.09	0.98	0.88	0.47	0.29	0.14	0.14	0.09
35-39	0.83	1.08	0.89	0.83	0.88	1.31	1.63	2.78	1.75	1.26	1.01	0.72	0.52	0.34	0.23	0.16	0.14
40-44	0.42	0.79	1.03	1.15	0.89	1.13	1.38	1.75	2.52	1.44	1.28	0.74	0.42	0.33	0.31	0.19	0.16
45-49	0.35	0.45	0.66	1.33	0.99	0.95	1.09	1.26	1.44	2.03	1.39	0.73	0.37	0.21	0.24	0.25	0.19
50-54	0.32	0.46	0.68	0.98	0.88	1.10	0.98	1.01	1.28	1.39	1.93	1.05	0.43	0.24	0.20	0.23	0.25
55-59	0.40	0.47	0.46	0.62	0.59	0.86	0.88	0.72	0.74	0.73	1.05	1.60	0.63	0.33	0.19	0.14	0.23
60-64	0.31	0.30	0.22	0.30	0.27	0.39	0.47	0.52	0.42	0.37	0.43	0.63	1.07	0.43	0.33	0.12	0.14
65-69	0.21	0.28	0.23	0.15	0.17	0.20	0.29	0.34	0.33	0.21	0.24	0.33	0.43	0.90	0.35	0.17	0.12
70-74	0.09	0.19	0.19	0.19	0.10	0.13	0.14	0.23	0.31	0.24	0.20	0.19	0.33	0.35	0.68	0.23	0.17
75-79	0.12	0.16	0.24	0.18	0.09	0.09	0.14	0.16	0.19	0.25	0.23	0.14	0.12	0.17	0.23	0.40	0.23
80+	0.12	0.12	0.16	0.24	0.18	0.09	0.09	0.14	0.16	0.19	0.25	0.23	0.14	0.12	0.17	0.23	0.40

Table S1. The contact matrix from [23], after preprocessing as described in Section 3.1.

50%. (We refer to these as very strict mitigations, strict mitigations, and moderate mitigations.) Note that the panel for $R_0 = 2.7$ with strict mitigations (70% reduction) includes the scenarios discussed in Section 4.2.

In each of these figure panels, scenarios where peak ICU utilization does not exceed the nominal ICU capacity by more than 50% are highlighted. Note that this includes some scenarios which exhibit a large second wave and thus exhibit many mortalities.

The figures for these scenarios all plot 36 months of simulation. In a few cases, a small but non-negligible part of the second wave occurs past this point. Mortality and ICU utilization for each scenario is summarized in Table S2; this data is based on a 10 year simulation period, to capture second waves extending beyond 36 months.

We examine the sensitivity of our analysis to qualitative choices in Fig S5. Here we consider scenarios where children or young adults are 50% less susceptible and/or 90% less infectious, as well as a situation where the infectious compartment is divided into sequential compartments, so that the ODE model corresponds to the case the infectious period of a random individual is gamma- rather than exponentially-distributed, with shape parameter $k = 5$.

S4 Full model description

In addition to the vector-valued compartments S, I, and R, our full model uses two additional (vector) compartments C' and C, corresponding to the populations who are infected and will enter ICU critical care, and those are are currently in ICU critical care, respectively. Letting ζ be a vector giving the rates at which people in each age group are admitted to the ICU, the differential equation model is elaborated as follows and as

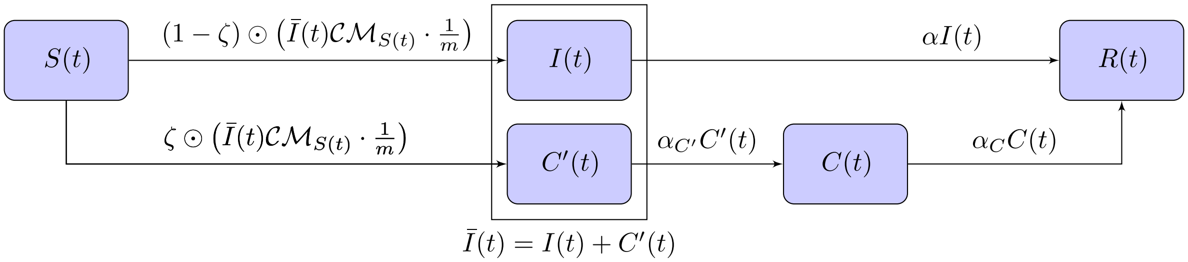

Fig S4. Schematic of the full differential equation model. The vector valued compartments $S(t), I(t), R(t), C'(t), C(t)$ represent the susceptible, infected, recovered/deceased, pre-ICU care, and ICU care populations, respectively, as population vectors where each coordinate cooresponds to the population in a single age group. Of the susceptible individuals who become infected, the ICU-rate-by-age vector ζ determines which individuals will enter the pre-critical compartment $C'(t)$ instead of $I(t)$. Individuals in $C'(t)$ proceed to the critical-care population $C(t)$ after an average of $\frac{1}{\alpha_{C'}}$ days, and then proceed to $R(t)$ after $\frac{1}{\alpha_C}$ days.

illustrated in Fig S4:

$$\frac{dI(t)}{dt} = (1-\zeta)\odot\left((I(t)+C'(t))\mathcal{CM}_{S(t)}\cdot\frac{1}{m}\right) - \alpha I(t), \tag{6}$$

$$\frac{dS(t)}{dt} = -(I(t)+C'(t))\mathcal{CM}_{S(t)}\cdot\frac{1}{m} \tag{7}$$

$$\frac{dR(t)}{dt} = \alpha I(t) + \alpha_C C(t). \tag{8}$$

$$\frac{dC'(t)}{dt} = \zeta\odot\left(I(t)\mathcal{CM}_{S(t)}\cdot\frac{1}{m}\right) - \alpha_{C'}C'(t) \tag{9}$$

$$\frac{dC(t)}{dt} = \alpha_{C'}C'(t) - \alpha_C C(t) \tag{10}$$

Here $\alpha_{C'} = 1/4$ and $\alpha_C = 1/10$. Conceptually, individuals which will eventually be admitted to the ICU (at relative rates, by age, given by the vector ζ) transit from S not to I but instead to C'. After an average of $1/\alpha_{C'}$ days, they enter the ICU compartment C, and then after $\frac{1}{\alpha_C}$ days, the proceed to the compartment R. For our parameter choices these periods are 4 and 10 days respectively. Note that the second parameter choice, which we take as a weighted average from [20], has a direct effect on the overall levels of ICU utilization that we report. The first parameter choice of 4 days has essentially no effect on our results; although individuals remain part of the transmitting population while in the compartment C', this population is so small compared to the rest of the population that lengthening or shortening this 4 day period would not meaningfully affect the dynamics (except to shift by a corresponding number of days the period of ICU utilization).

	R0	strategy	supression	age	fraction	mortalities	peak.ICU
1	2.40	homogeneous	optimal	NA	NA	6.36	2.92
2	2.40	heterogeneous	90%	up to 39	0.67	9.23	5.18
3	2.40	heterogeneous	90%	up to 49	0.67	7.35	2.89
4	2.40	heterogeneous	90%	up to 59	0.67	5.84	1.26
5	2.40	heterogeneous	90%	up to 39	1	1.24	0.88
6	2.40	heterogeneous	90%	up to 49	1	1.87	1.70
7	2.40	heterogeneous	90%	up to 59	1	3.72	3.80
8	2.40	heterogeneous	70%	up to 39	0.67	7.83	2.54
9	2.40	heterogeneous	70%	up to 49	0.67	5.74	1.24
10	2.40	heterogeneous	70%	up to 59	0.67	4.32	1.95
11	2.40	heterogeneous	70%	up to 39	1	3.12	2.37
12	2.40	heterogeneous	70%	up to 49	1	4.07	3.61
13	2.40	heterogeneous	70%	up to 59	1	5.69	5.61
14	2.40	heterogeneous	50%	up to 39	0.67	5.02	2.41
15	2.40	heterogeneous	50%	up to 49	0.67	5.08	3.10
16	2.40	heterogeneous	50%	up to 59	0.67	5.82	3.87
17	2.40	heterogeneous	50%	up to 39	1	5.62	4.44
18	2.40	heterogeneous	50%	up to 49	1	6.44	5.77
19	2.40	heterogeneous	50%	up to 59	1	7.66	7.47
20	2.70	homogeneous	optimal	NA	NA	7.03	3.38
21	2.70	heterogeneous	90%	up to 39	0.67	10.26	6.35
22	2.70	heterogeneous	90%	up to 49	0.67	7.87	3.10
23	2.70	heterogeneous	90%	up to 59	0.67	6.03	1.44
24	2.70	heterogeneous	90%	up to 39	1	1.45	1.21
25	2.70	heterogeneous	90%	up to 49	1	2.15	2.22
26	2.70	heterogeneous	90%	up to 59	1	4.11	4.80
27	2.70	heterogeneous	70%	up to 39	0.67	8.22	2.31
28	2.70	heterogeneous	70%	up to 49	0.67	5.66	2.03
29	2.70	heterogeneous	70%	up to 59	0.67	4.33	3.01
30	2.70	heterogeneous	70%	up to 39	1	3.81	3.32
31	2.70	heterogeneous	70%	up to 49	1	4.78	4.80
32	2.70	heterogeneous	70%	up to 59	1	6.45	7.19
33	2.70	heterogeneous	50%	up to 39	0.67	5.78	3.82
34	2.70	heterogeneous	50%	up to 49	0.67	6.33	4.68
35	2.70	heterogeneous	50%	up to 59	0.67	7.10	5.63
36	2.70	heterogeneous	50%	up to 39	1	6.79	6.14
37	2.70	heterogeneous	50%	up to 49	1	7.55	7.67
38	2.70	heterogeneous	50%	up to 59	1	8.75	9.64
39	3.00	homogeneous	optimal	NA	NA	7.53	4.35
40	3.00	heterogeneous	90%	up to 39	0.67	11.14	7.32
41	3.00	heterogeneous	90%	up to 49	0.67	8.37	3.29
42	3.00	heterogeneous	90%	up to 59	0.67	6.25	2.00
43	3.00	heterogeneous	90%	up to 39	1	1.77	1.56
44	3.00	heterogeneous	90%	up to 49	1	2.40	2.74
45	3.00	heterogeneous	90%	up to 59	1	4.45	5.76
46	3.00	heterogeneous	70%	up to 39	0.67	8.46	2.00
47	3.00	heterogeneous	70%	up to 49	0.67	5.26	2.92
48	3.00	heterogeneous	70%	up to 59	0.67	5.10	4.15
49	3.00	heterogeneous	70%	up to 39	1	4.48	4.33
50	3.00	heterogeneous	70%	up to 49	1	5.44	6.02
51	3.00	heterogeneous	70%	up to 59	1	7.13	8.74
52	3.00	heterogeneous	50%	up to 39	0.67	7.00	5.40
53	3.00	heterogeneous	50%	up to 49	0.67	7.51	6.38
54	3.00	heterogeneous	50%	up to 59	0.67	8.26	7.50
55	3.00	heterogeneous	50%	up to 39	1	7.89	7.91
56	3.00	heterogeneous	50%	up to 49	1	8.59	9.59
57	3.00	heterogeneous	50%	up to 59	1	9.74	11.79

Table S2. Mortalities and ICU admissions by scenario. Like homogeneous strategies, heterogeneous strategies can perform suboptimally because they are too relaxed and allow too large of an epidemic in the mitigation period, or too strict and invite a second wave in the hypothetical resumption normal transmission levels. Highlighted rows are scenarios which have lower mortalities than the optimal homogeneous measures for the same R_0.

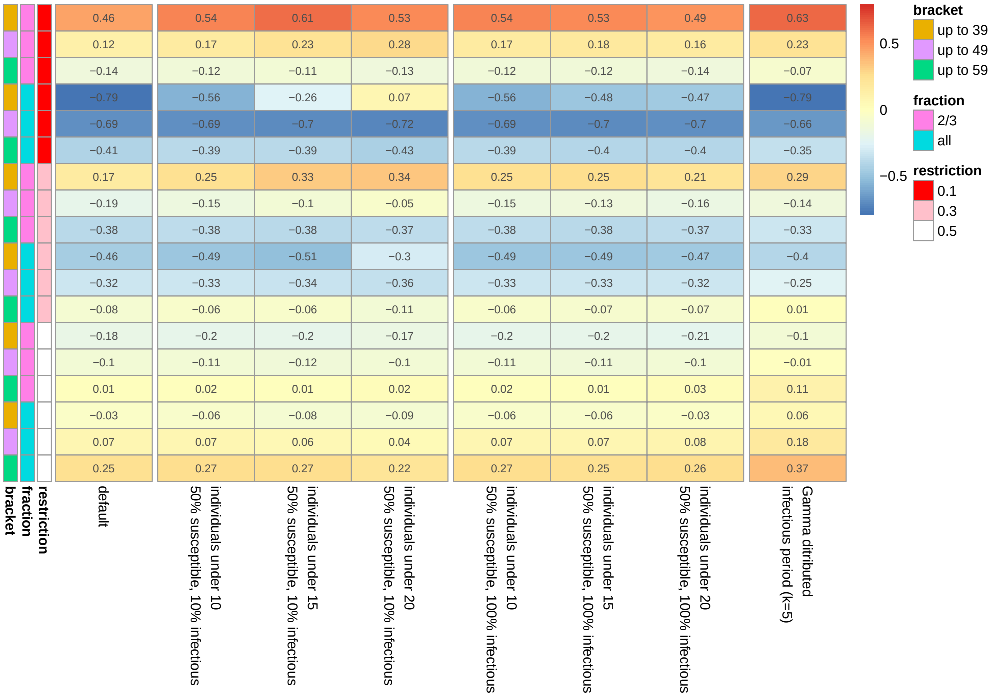

Fig S5. For various qualitative changes to our model represented by columns in this table (i.e., to the transmission characteristics of children, or to the distribution of the infectious period) we compare, in each case, the mortality reduction (indicated by negative numbers) achieved by the heterogeneous strategies, which each correspond to a row of the table. Note that the 3 color-bars on the left indicate the heterogeneous strategy corresponding to the row, according to the legend. There is broad agreement across these qualitative variants that strong reductions in mortality can be achieved with heterogeneous strategies. For example, releasing 2/3 of people under 60 with 70% mitigations on the rest (restriction=.3) achieves a reduction of between 33% and 38% for all these scenarios, compared with optimum homogeneous measures. Note that each qualitative variant is compared against optimum homogeneous measures for the same regime.

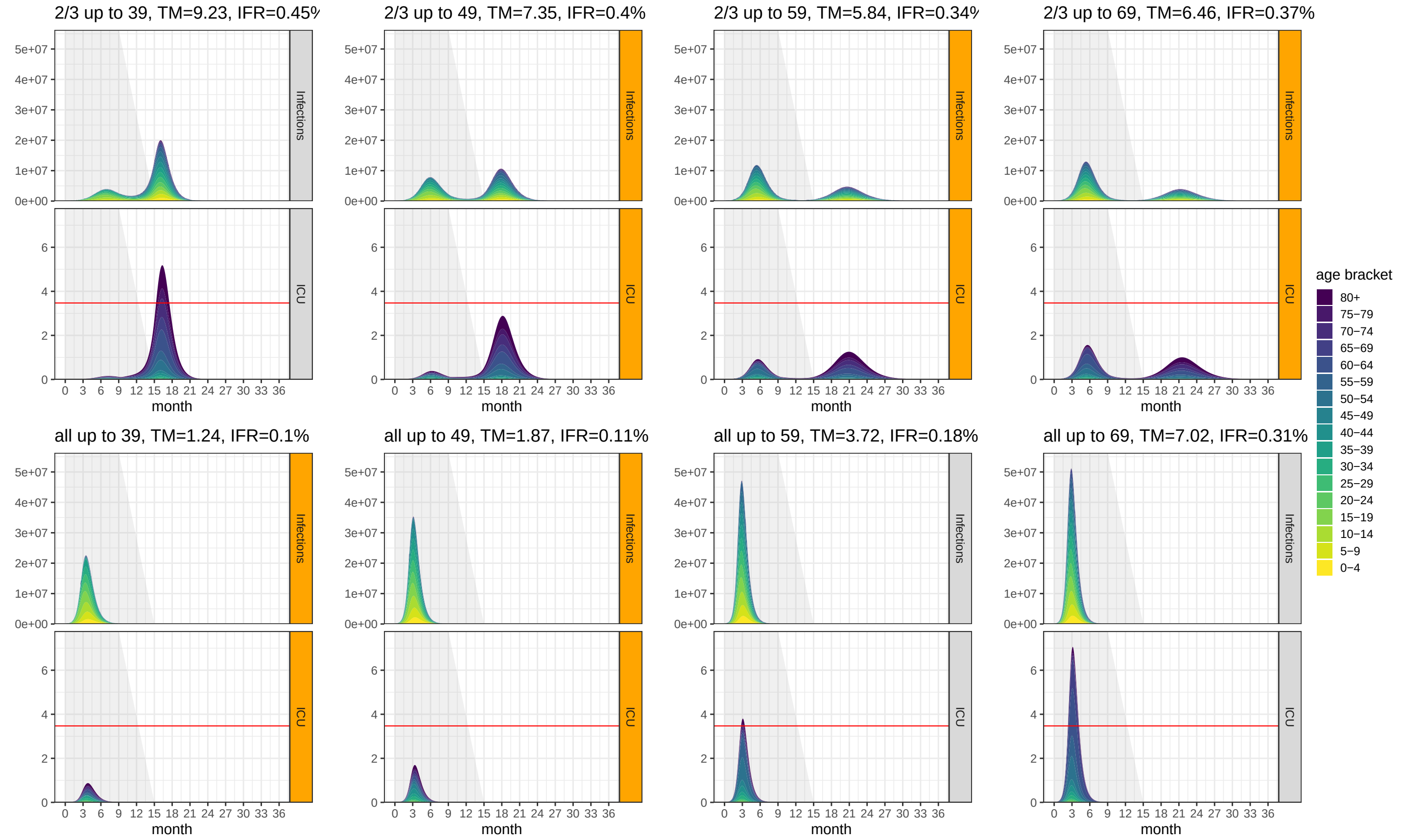

Fig S6. Scenarios for $R_0 = 2.4$, very strict mitigations (90% reduction in transmission for groups subject to mitigations). Scenarios which do not exceed the nominal ICU capacity by more than 50% are highlighted.

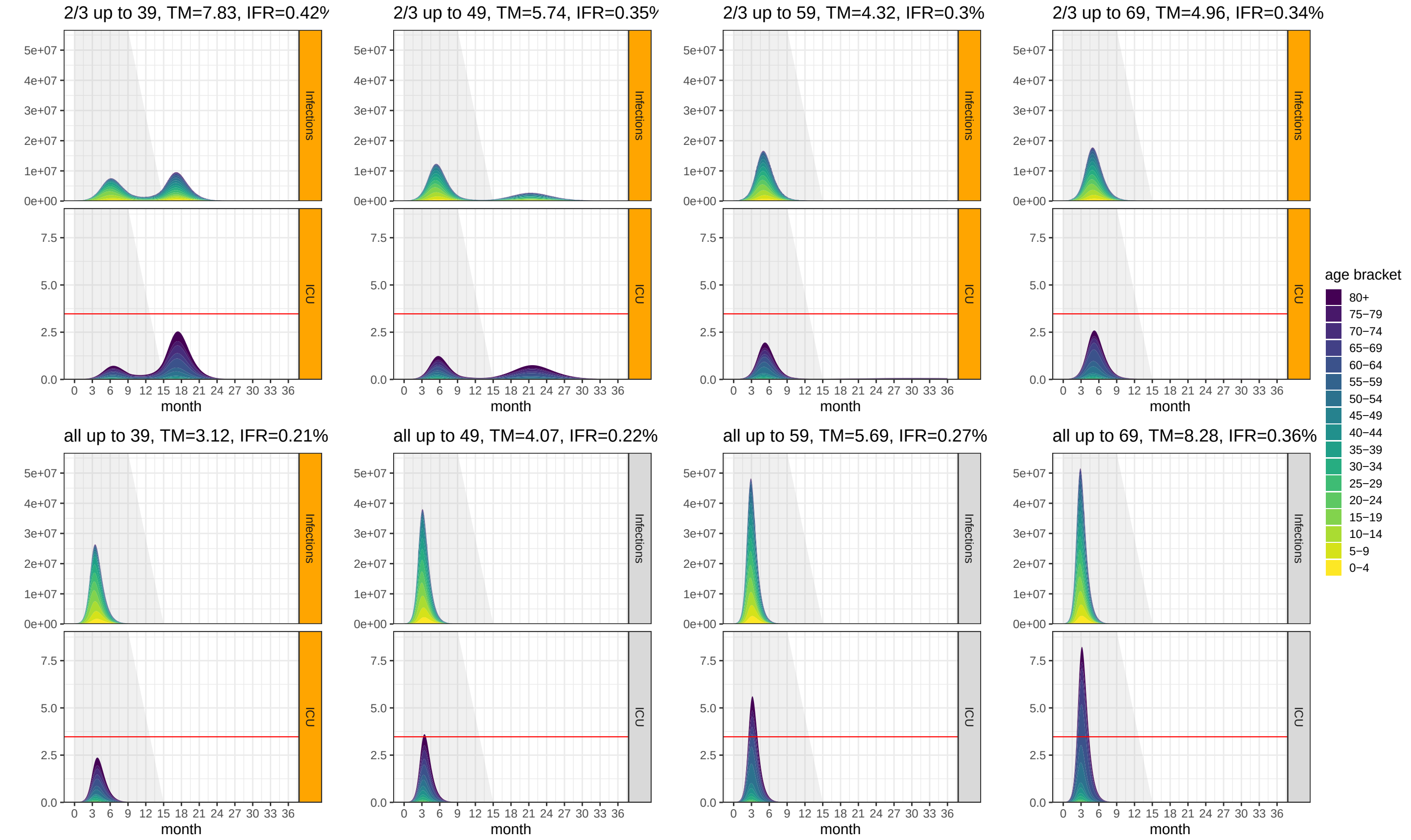

Fig S7. Scenarios for $R_0 = 2.4$, strict mitigations (70% reduction in transmission for groups subject to mitigations). Scenarios which do not exceed the nominal ICU capacity by more than 50% are highlighted.

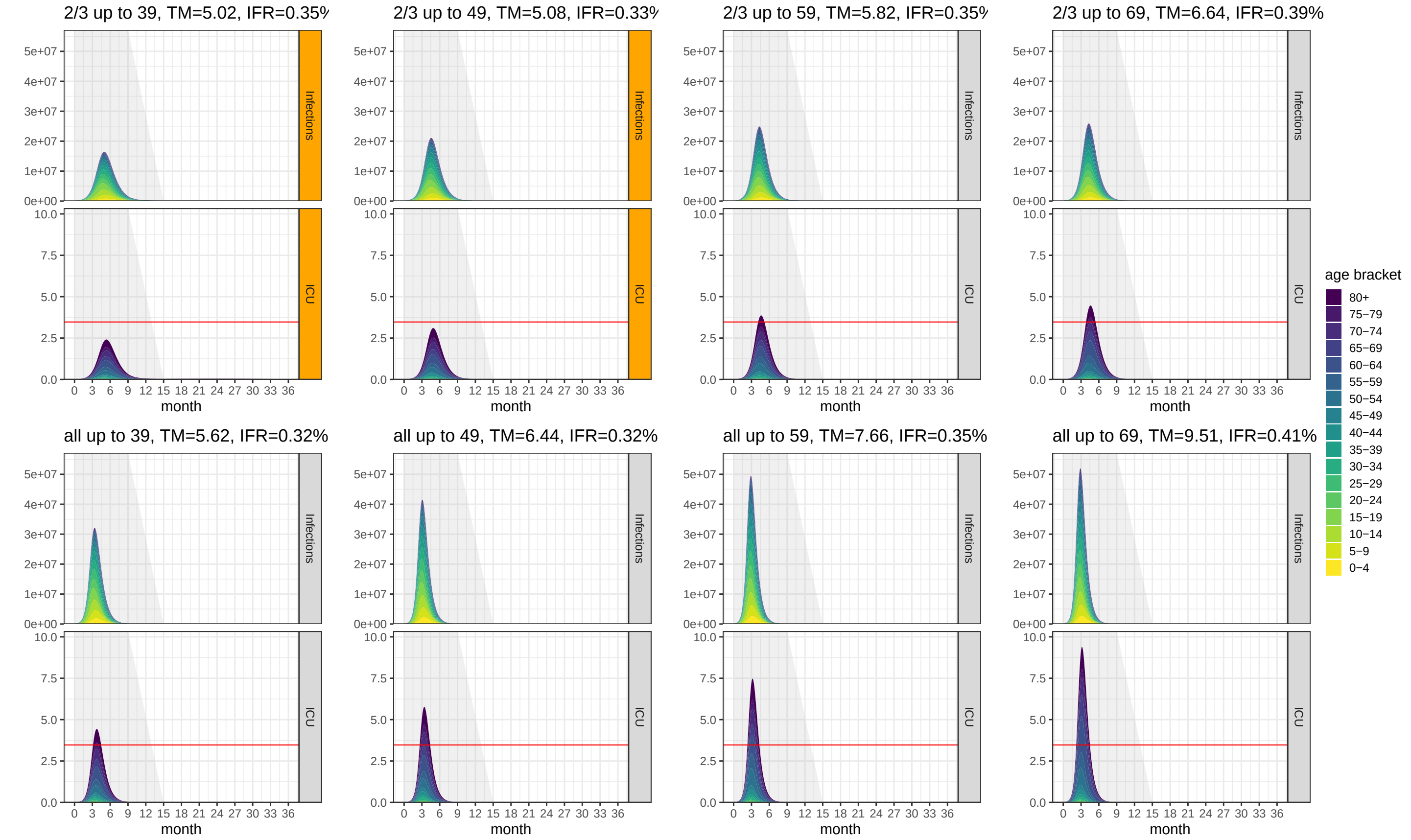

Fig S8. Scenarios for $R_0 = 2.4$, moderate mitigations (50% reduction in transmission for groups subject to mitigations). All of these scenarios exceed the nominal ICU capacity by more than 50%.

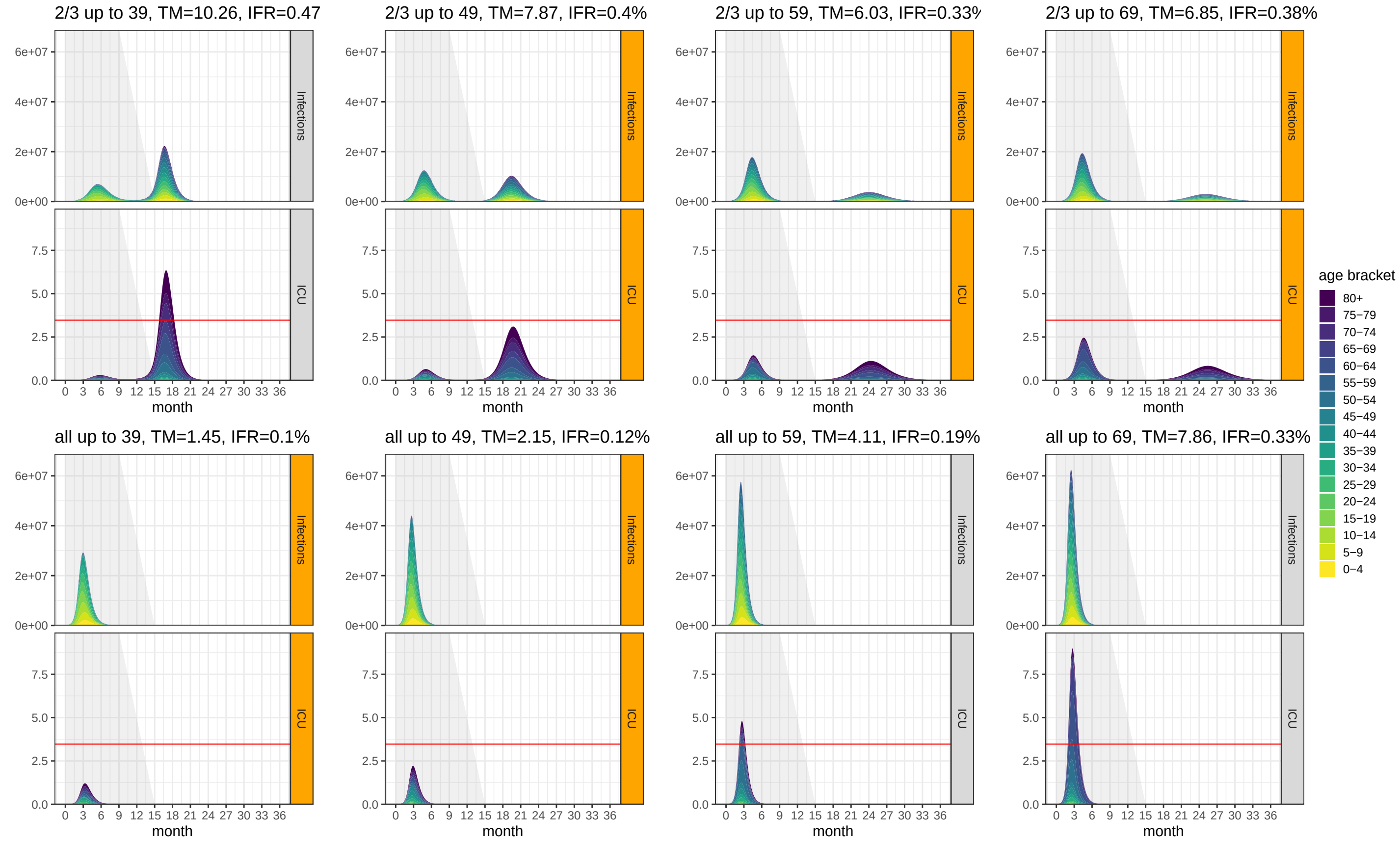

Fig S9. Scenarios for $R_0 = 2.7$, very strict mitigations (90% reduction in transmission for groups subject to mitigations). Scenarios which do not exceed the nominal ICU capacity by more than 50% are highlighted.

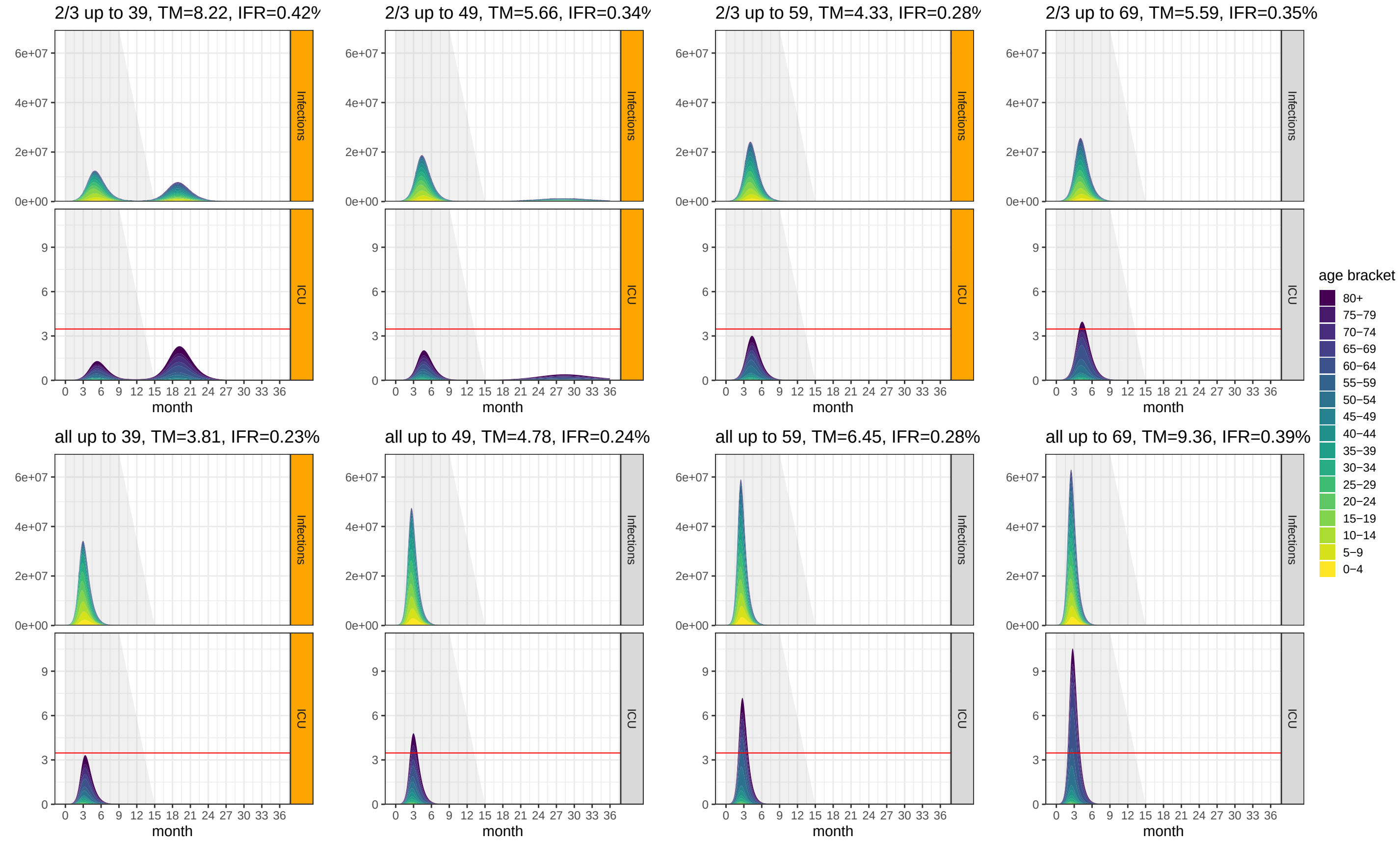

Fig S10. Scenarios for $R_0 = 2.7$, strict mitigations (70% reduction in transmission for groups subject to mitigations). Scenarios which do not exceed the nominal ICU capacity by more than 50% are highlighted.

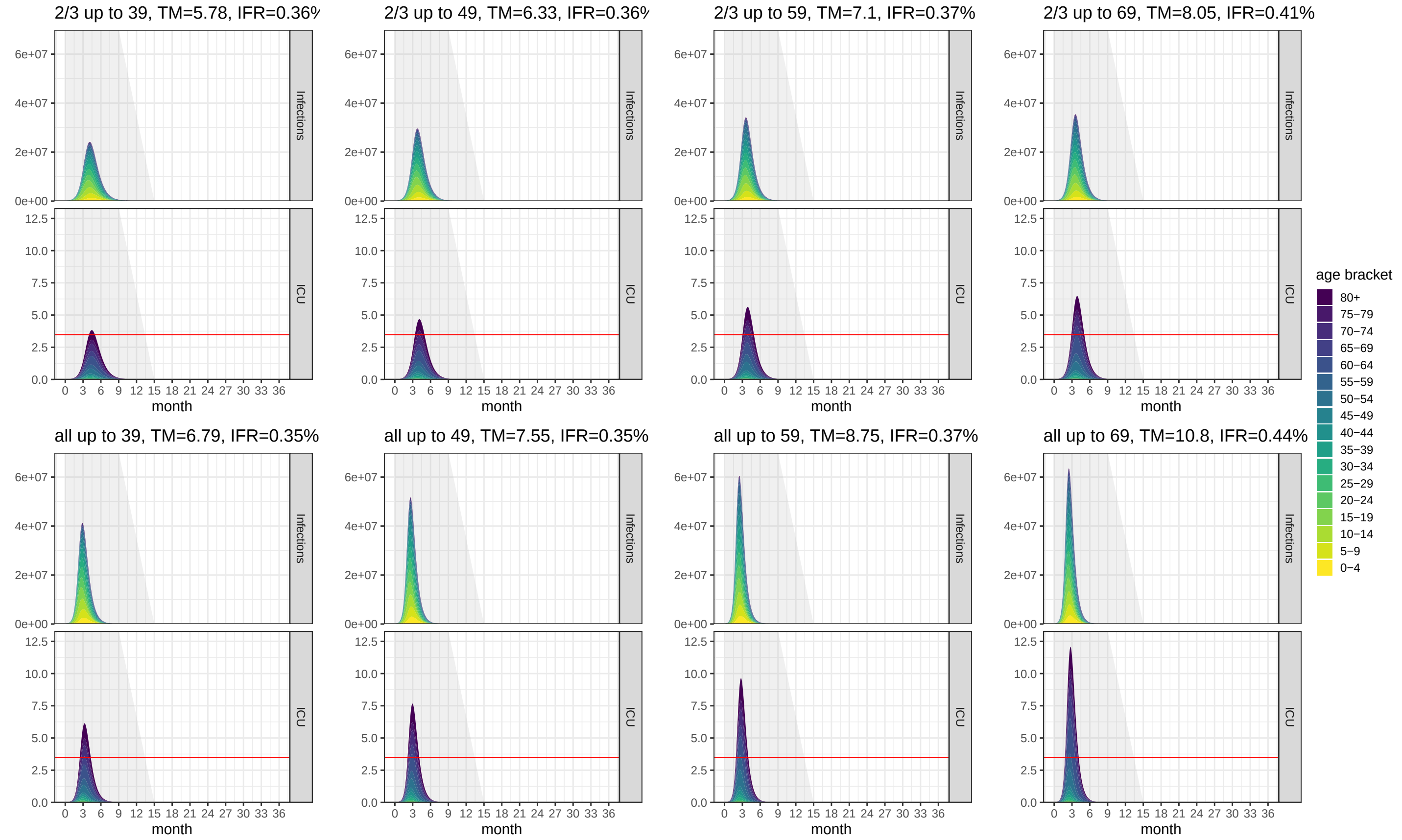

Fig S11. Scenarios for $R_0 = 2.7$, moderate mitigations (50% reduction in transmission for groups subject to mitigations). All of these scenarios exceed the nominal ICU capacity by more than 50%.

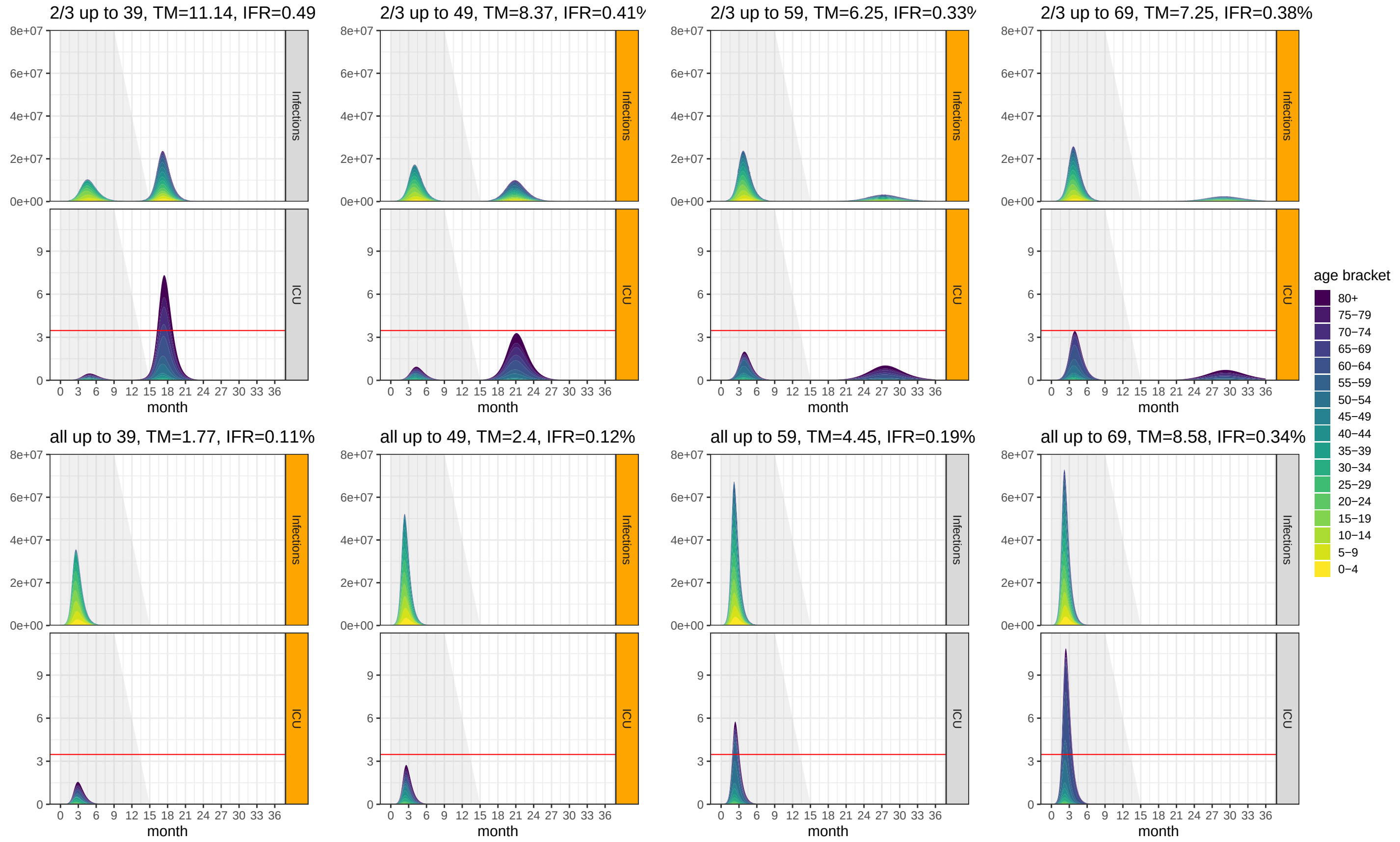

Fig S12. Scenarios for $R_0 = 3.0$, very strict mitigations (90% reduction in transmission for groups subject to mitigations). Scenarios which do not exceed the nominal ICU capacity by more than 50% are highlighted.

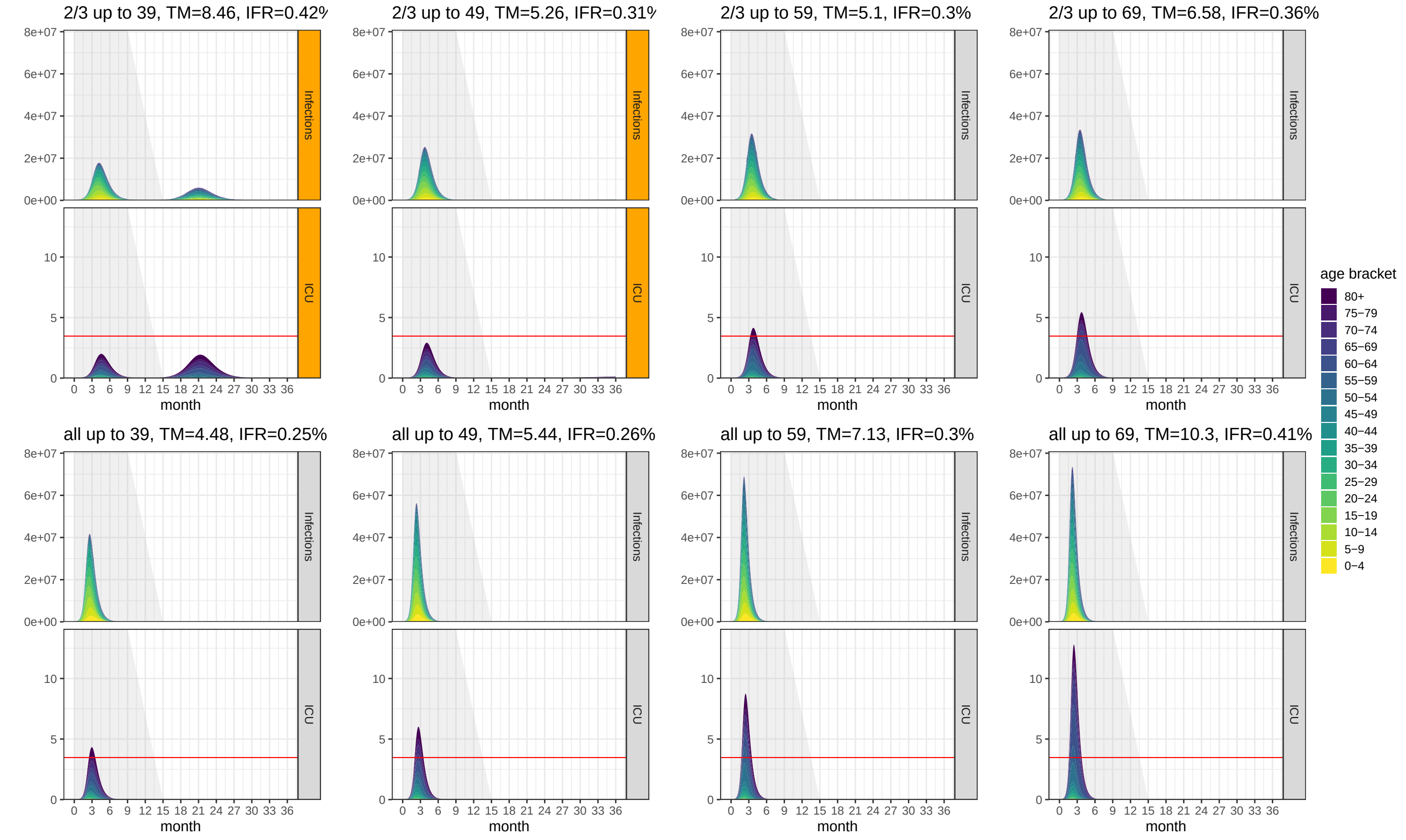

Fig S13. Scenarios for $R_0 = 3.0$, strict mitigations (70% reduction in transmission for groups subject to mitigations). Scenarios which do not exceed the nominal ICU capacity by more than 50% are highlighted.

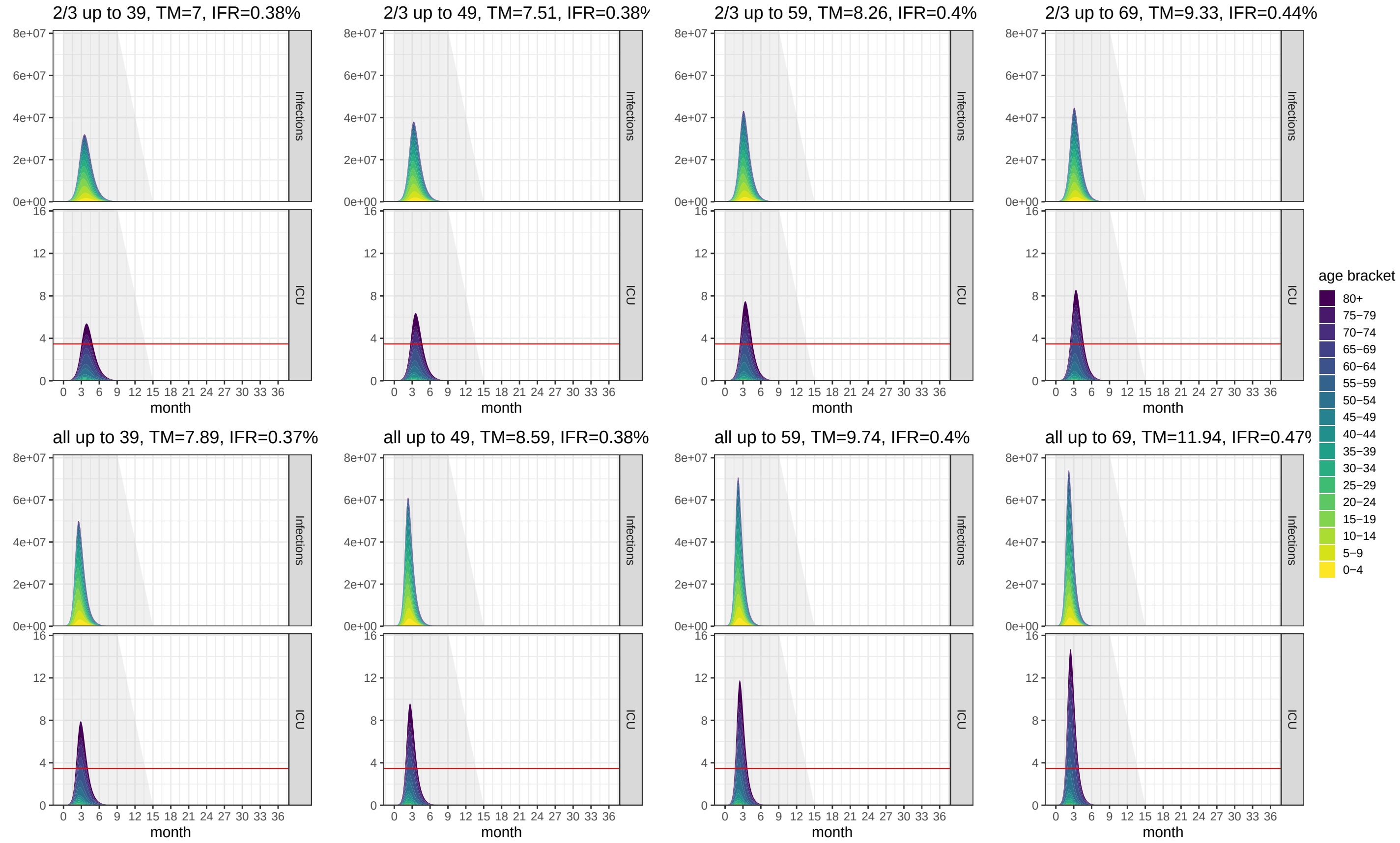

Fig S14. Scenarios for $R_0 = 3.0$, moderate mitigations (50% reduction in transmission for groups subject to mitigations). All of these scenarios exceed the nominal ICU capacity by more than 50%.
