# Supplementary material for: Modeling strict age-targeted mitigation strategies for COVID-19
Source: PLoS One. 2020 Jul 24;15(7):e0236237. doi: 10.1371/journal.pone.0236237 (PMC7380601; doi:10.1371/journal.pone.0236237)
Supplement: Attachment — Submitted filename: PLOS One reply.pdf [file pone.0236237.s003.pdf]

Review response:

Below we respond to reviewers remarks point by point. In many cases we have made additions to the main text or to the SI in response to suggestions. We have also made the following changes to the manuscript, apart from those requested by referees:

Motivation section

We have added a section on motivation to explain what is qualitatively significant about our findings beyond the intuitive notion that protecting at-risk populations should reduce mortality. In particular, the key contribution is the qualitative point that targeted mitigations can greatly reduce mortality even if mitigations eventually end; this finding, as we discuss in this section, depends on the anticorrelation of COVID mortality rates and contact patterns by age.

Section on children transmission

There has been discussion in the literature recently on the transmission potential of young children with respect to COVID (in particular, it has been suggested that young children may be less susceptible and/or less infectious when infected than adults). Because younger individuals play an outsize role in the contact matrix, it would be reasonable to wonder whether these results would have a large mitigating effect on our findings. In particular, one could worry that if the younger population contributed less than expected to early epidemic growth because of lower transmissibility, that the transmission potential of the older population may be underestimated based on the approach of using traditional contact matrices with estimated R_0. In the new section we discuss these issues and examine the sensitivity of our method to these questions.

Contact matrix granularity

The contact matrix provided by [22] gives contact patterns by age groups in age-buckets of 5 years. In our previous version, we collapsed these buckets to 10-year groupings. In our new revision we keep the original 5-year bucketing for the contact matrix. (When examining transmission from/to children in the new section, this change allows us to consider scenarios for children under 10 and also under 15.)

A note on comparing results between versions

Our revision makes three changes which affect our mortality and ICU figures. The first change is using contact patterns by the original 5-year age buckets as originally reported in [22], instead of collapsing to 10-year age buckets as we did in our first version. Because using 5-year buckets results in modeling slightly more of the actual contact pattern heterogeneity, this results in slightly decreased estimates of attack rates.

On the other hand, in response to a comment by Review 3, we have updated our population data to be Census estimates from 2019; previously we were using data from the last completed official Census, in 2010. This change means we are using a slightly larger overall population than before, and with a slightly different (and generally, older) age distribution.

Finally, also in response to Reviewer 3, we have updated our modeling of ICU utilization. We now take our parameters for ICU utilization from the recent hospitalization study [20], giving an average ICU stay length of 10 days in our case. Since this is shorter than the 14 day period we used previously, this results in slightly lower estimates of ICU utilization overall in the new revision.

We also now report an IFR for each scenario, to emphasize the role of shifting the risk-profile of the infected population on the mortality reductions observed in the scenarios we model.

In all, the following figures (numbering from the new revision) are new or have substantial changes:
Figure 1 (additional panels added to righthand side)
Figure 8
Figure 9
Table S1
Figure S1,S2,S3,S4
Figure S5

The remaining figures have been remade with the updated 2019 population data, more detailed contact matrix, and updated ICU model, but otherwise are unchanged.

We have also updated our notation to be more consistent with convention so that the compartment R includes the population which has died (i.e., now M is a subset of R, rather than disjoint from it).

Journal comments

Journal Requirements:

When submitting your revision, we need you to address these additional requirements.

We have updated formatting to use the PLOS style template.

2. Thank you for stating the following financial disclosure:

"The funders had no role in study design, data collection and analysis, decision to

publish, or preparation of the manuscript"

At this time, please address the following queries:

a) Please clarify the sources of funding (financial or material support) for your study. List the grants or organizations that supported your study, including funding received from your institution.

b) State what role the funders took in the study. If the funders had no role in your study, please state: “The funders had no role in study design, data collection and analysis, decision to publish, or preparation of the manuscript.”

c) If any authors received a salary from any of your funders, please state which authors and which funders.

d) If you did not receive any funding for this study, please state: “The authors received no specific funding for this work.”

> Please include your amended statements within your cover letter; we will change the online submission form on your behalf.

We have addressed this in our cover letter.

3. We note that a table in your submission may be copyrighted, this is noted with the following text: "The mortality rate and rate of ICU admissions per infection are taken from Report 9 of the team at Imperial College London [4]; we use the data from their Table 3".

…

We have changed how we display Table 1. Now, instead of reproducing the table from the Imperial report verbatim, we display a table of the same data but with our own headings. Because we are just showing data (which is used in our model) and not the original annotation or curation of the data, we do not believe copyright applies here. See, e.g., https://www.lib.sfu.ca/help/academic-integrity/copyright/data-copyright

4. Please ensure that you refer to Figure 1 in your text as, if accepted, production will need this reference to link the reader to the figure.

Reviewer #1:

Thank you for your comments. As we say in our manuscript, our desire is to use a simple model to demonstrate an important qualitative feature of the COVID-19 epidemic and it's potential implication for mitigation efforts, which stems from the fact that, by age, natural contact patterns and the COVID mortality rage are strongly anti-correlated.

Below we respond point by point to your list of criticisms, indicating for some that we have added analyses to the SI to demonstrate robustness to these kinds of changes, and for others that these critiques would undermine almost any (and certainly, many recent high profile) efforts to model the COVID-19 epidemic. (We have combined responses for identical or very similar critiques.)

As a general point, we agree that a more complex model may be desired if our goal was to predict precise numbers of deaths which will occur at specific timepoints in the future. However, in our manuscript we explicitly disclaim this goal in favor of aiming to demonstrate an important qualitative point; which is that the anticorrelation by age between mortality rates and contact natural patterns can have a very large effect on COVID-19 mortality.

To make this qualitative point, a simpler model makes our analysis more transparent, with less room for overfitting or for speculative parameter selection to allow us to exaggerate our findings. As a comparison point, the recent high-profile article [24] (as cited in our revision: Prem et al, The effect of control strategies to reduce social mixing on outcomes of the COVID-19 epidemic in Wuhan, China: a modelling study. *The Lancet Public Health*) also did age-sensitive modeling, without any of the additional complexities described in the points below.

1. Behavior changes with the illness when symptoms appear.
2. Behavior changes with the social perception of risk in an epidemic.

Modeling these additional complexities would introduce more speculation and freedom to freely choose parameters and obscure the essential qualitative point our paper is making. Note that [24] also does not model things like isolation (and certainly not social perception of risk).

3. The course of SARS-CoV-2 changes with age. In particular, recovery times.
5. The recovery time that matters is the time from onset of contagiousness to isolation or end of the contagious period (whatever comes first). Such times depend on age.

We have added the citation and explanation below to the manuscript:
While disease course should vary by age, a large-scale study in Israel found recovery periods between 13 and 15 days for all age groups [citation].

4. It is suspected that mild cases are less contagious than severe cases (before isolation).

The only way in which our compartmental model tracks severe cases separately is through the ICU-related compartments. These are too small as a fraction of the total population for the transmission model attached to them to have large quantitative effects. For example the recent paper [17] (Kissler et all, Projecting the transmission dynamics of SARS-CoV-2 through the post pandemic period, *Science*) uses a similar compartmental model to ours, distinguishing infected persons only by hospitalization/critical care status.

If the suggestion is that our modeling should incorporate additional heterogeneity in the population; for example, with gradations of disease severity outside of the model of hospitalization, then this seems like an interesting project but not necessary to demonstrate the large qualitative effect of age-targeted mitigations which is the point of our paper (and also not done by [17],[24], etc).

If the suggestion is that younger cases may be more mild and thus less contagious, then this is a concern addressed by our new section on modified assumptions for susceptibility and infectiousness of children.

6. The contagious period is not exponentially distributed (without exponentially distributed times for each compartment, there is no support for ODE models)
8. A homogeneous contact (without social structure) limits any model to small communities.
9. An ODE approach limits its scope to large numbers in each compartment.
With the exception of the question about the exponentially distributed contagious period, these points argue against the general use of ODE based compartmental models. But their use is still common, including among high profile papers on the COVID epidemic (such as [17] and [23]). There is an inherent tradeoff in model complexity, especially for a new epidemic like COVID subject to significant parameter uncertainty. Simple models allow transparent demonstrations of important qualitative phenomena. It is of course perfectly possible to use ODE models of non-exponentially distributed---e.g., Erlang (gamma) distributed---infectious periods. In general this additional complexity is most relevant when periodicity from seasonal or other periodic forcing is present. Note that [17], which includes this modeling detail, is modeling seasonal forcing, while [24] (like us) is not modeling periodic effects, and does not include this detail.

Nevertheless, in the new SI section 3 (Sensitivity of analysis), we demonstrate that our qualitative results still hold when modeling an Erlang (gama) distribution for the infectious period.

7. Social contact at normal times is not the important kind of contact in terms of the propagation of the epidemic. What is relevant is the ability to transmit the illness.
10. The combination of (6) and (7) may limit the scope to the empty set.

One aim of our approach is to identify mortality in various scenarios under the assumption that eventually, relative social contact rates will return to near-normal levels. Note also that the contact matrices we use in our paper are explicitly produced for the purpose of modeling infectious diseases, and frequently used for this purpose, as in [23].

We have added a paragraph to the introduction to more clearly emphasize the point of our paper early on, which explains the relevance of modeling normal contact patterns:

Note that while it is not at all surprising that targeting mitigations at higher risk groups can greatly reduce mortality during mitigations, our main contribution is to emphasize that this result still holds even if transmission rates for all groups are eventually relaxed. In particular, as we discuss in Section \ref{s.Contact}, the fact that this holds for COVID-19 depends on the coincidence that age-specific mortality rates for COVID are very strongly anti-correlated with age-specific contact patterns during periods of normal social interaction.

12. Do people in the USA continue with their social-contacts being active epidemiologically when hospitalized? I would really be surprised. This is just another feature the authors built in their model without realizing it. It is the consequence of the faulty epistemology.

Our manuscript does not claim this. We have added discussion of this to the new section on the ICU model:

Since the number of ICU patients is very small compared with the rest of the population, our results are insensitive to the precise model of transmission attached to ICU patients...

Nevertheless, in response to your request, our modeling code now removes the ICU population from the transmission population.

11. R0 is model depending. As such, it cannot be read from the data.

We have changed the wording from “empirical” to “reported”.

I did not read beyond section 3.

Reviewer #2:

In the paper, the authors proposed a SIR-like epidemic model with contact matrix and study the effect of age-targeted mitigation strategies. It is an innovation point of the manuscript. Results

are interesting and satisfactory. There are, however, still some minor problems need to be solved before publication.

1. Please give the exact value of contact matrix C when modeling mitigation strategies.
We do this now in the supplement. Table S1 shows the precise numerical values of the original contact matrix after processing. Figure S1 describes the precise arithmetic change to the matrix for each mitigation strategy, and illustrates the result.

2. Please give the clear description of mitigation strategies
In S 4.2:
In each of these scenarios, depicted in Figures 4, 5, and 6, we assume that the relaxed population is subject to normal transmission levels, while transmission to, from, and within the rest of the population is depressed by 70% from normal levels.
3. The figures are unclear, especially the figure of ICU.
We have added a comment to each caption of the first two of these figures clarifying that the units for the ICU graph are beds required per 10,000 individuals in the overall population.
4. I cannot understand the result of figure 2 B. Why there are no infections at the beginning?
During containment, the number of infections is kept very small (so that on the scale of the figure, it is not visually perceptible). Once mitigations end, exponential growth takes place and we eventually see an epidemic.

Reviewer #3:

In the present work, the authors present a model for a strict age-targeted mitigation strategies for COVID-19. The model is based on a standard SIR model adapted to include an aged specific contact matrix. Also, some age-specific epidemiological parameters were included in the model. The author show how such a strategy can avoid the collapse of the ICU units as the contagion of the elderly is smooth and even lower than in the absence of such a strategy. In turn, the targeted isolation can make the quarantine more tolerable for the rest of the population. The main results are in part trivial, as a natural result of partially isolation part of a population is preventing them from being infected. In order for this model to prove of some utility would be if it can provide robust qualitative results.

We have added a new section to the introduction (Motivation) to explain better what underlies the main contribution and why it goes beyond showing simply that reducing transmission to at-risk populations reduces mortality. We have also added the following clarifying paragraph to the introduction:

Note that while it is not at all surprising that targeting mitigations at higher risk groups can greatly reduce mortality during mitigations, our main contribution is to emphasize that this result still holds even if transmission rates for all groups are eventually relaxed. In particular, as we discuss in Section 2, the fact that this holds for COVID-19 depends on the coincidence that

age-specific mortality rates for COVID are very strongly anti-correlated with age-specific contact patterns during periods of normal social interaction.

The model uses a contact matrix that is asymmetric due to the methodology used to build it. The results are based in a directed survey, where there is always a pointing and a pointed person. This is the origin of the asymmetry and not because they correspond to frequencies of interactions. It is not clear how the authors build their symmetric matrix. and where they got the information about the population pyramid.

We have clarified the wording in this section. You are right, of course, that the methodology for collecting the contact data introduces the asymmetry directly (since one contact may report an interaction while the other may not even be part of the survey). For the vector-valued SIR approach, the methodologically important way in which this introduces asymmetry is that it incorporates the effects of relative population sizes.. E.g., if one carried out a contact tracing experiment on just two groups, lefthanded and righthanded people, the result would be a highly asymmetric 2x2 matrix, with both right- and left-handed people reporting many more contacts with righthanded individuals than lefthanded ones. Since the matrix form of the SIR model we use already accounts for population distribution (via the vector S(t)), this population variation is divided out from the empirical data to give an estimate of the matrix of relative contact patterns.

This procedure is discussed, for example, on page 74 of the textbook *Epidemics: Models and Data using R*, (Springer) by Ottar N. Bjørnstad.

Note that the eigenvalue corresponding to R0 in our SIR model is not the eigenvalue of the symmetrized contact matrix C but instead of the matrix product C*diag(S(0)), which reverses the symmetrization procedure of correcting by population proportions.

(Note also that if this normalization step was done incorrectly, R0=1 would not be the epidemic threshold in our model, but our provided code makes it easy to verify that R0=1 is indeed the threshold.)

In our original manuscript we used population estimates from the 2010 U.S. Census. In our revision we indicate that we now use Age composition tables estimated by the Census as of 2019.

The dynamics of the ICU is not described. There is no accurate information about the permanence of patients in ICU units.

We have updated our ICU model and it's parameter choices, which we now take from the recent PNAS study which modeled ICU utilization for COVID [20]. The full model including the ICU arm is now described in detail in Section S4.

The prevalence of risk groups among the nonisolated population is not taking into account. This information is very relevant at the moment of an accurate estimation of the occupation of ICU. The information about the percentage of each age group ICU requirement is obtained from data collected from a different country, a different population. A simple research across reports from different countries show how scattered these percentages are.

It is of course correct that mortality rates and ICU utilization may vary for the United States (or other countries) than the source data we use, because of population-level differences. Unfortunately, detailed age-stratified U.S.-wide ICU utilization data is not currently available.

The qualitative findings of our study depend above all on the relative rates of mortality/morbidity by age group, which have been quite consistent across countries. For example, the largest-scale study to date on risk factors (*OpenSAFELY: factors associated with COVID-19-related hospital death in the linked electronic health records of 17 million adult NHS patients,* see Figure 3) found elevated risk for various conditions, but no common conditions like diabetes, high blood pressure, asthma, or obesity imparting significantly greater risk than sex or 10 additional years of age.

In the last weeks, we have seen a plethora of models, with a vast majority of them presenting contradicting and out of scale results.

In the present form, the status of the present model is conjectural only, with questionable robustness. It would be irresponsible to propose a public health policy on the basis of such a feeble analysis. The author should present a stronger and more founded model.

We hope the addition of the new checks and the “Sensitivity analysis” section of the SI helps to reassure that the broad qualitative conclusions we draw are not sensitive to reasonable choices for parameters made in our modeling. That said, we do **not** consider this manuscript as an argument in favor of a specific public health policy. We have emphasized this more clearly now in the discussion section:

It is important to emphasize, however, that the extent to which strategies like those considered here are the best approach depends on what other strategies are considered feasible. For example, if the COVID-19 epidemic can be contained indefinitely (i.e., if the second wave in Figure 1B can be avoided) then that approach will certainly prevent more COVID deaths. Our analysis shows, however, that if one believes population immunity will eventually play a role in ending the COVID-19 epidemic in some locations, then age-targeted strategies can have a large effect on the mortality incurred in reaching that population immunity.

We agree that the proliferation of models in recent weeks could easily give the impression that models have little agreement, producing vastly different numbers. However, on qualitative points the models are in excellent agreement. E.g., consider qualitative points like:

- The rate of early growth in an epidemic in different locations is well-correlated with final epidemic size in the absence of mitigations,
- Mitigations reducing transmission rates can have a significant effect on final epidemic size and total mortality,
- Lifting mitigations before a significant fraction of the population is immune (and without a robust system of contact tracing) is expected to lead to a second wave,
- etc.

The various modeling approaches are in wide agreement on these points, even if they disagree on precise quantitative predictions. Our contribution is to add another qualitative point to this list, namely:

- Exaggerating existing age-heterogeneous contact patterns can have a large effect on final mortality figures, even if normal contact patterns eventually resume.

And, like these other qualitative points, we are confident that this qualitative conclusion is robust to a wide range of parameter selection and modeling choices.
